# Supplementary figures and images for: Successful Treatment of Acute Uric Acid Nephropathy with Rasburicase in a Primary Central Nervous System Lymphoma Patient Showing a Dramatic Response to Methotrexate—Case Report
Source: J Clin Med. 2022 Sep 22;11(19):5548. doi: 10.3390/jcm11195548 (PMC9571497; doi:10.3390/jcm11195548)

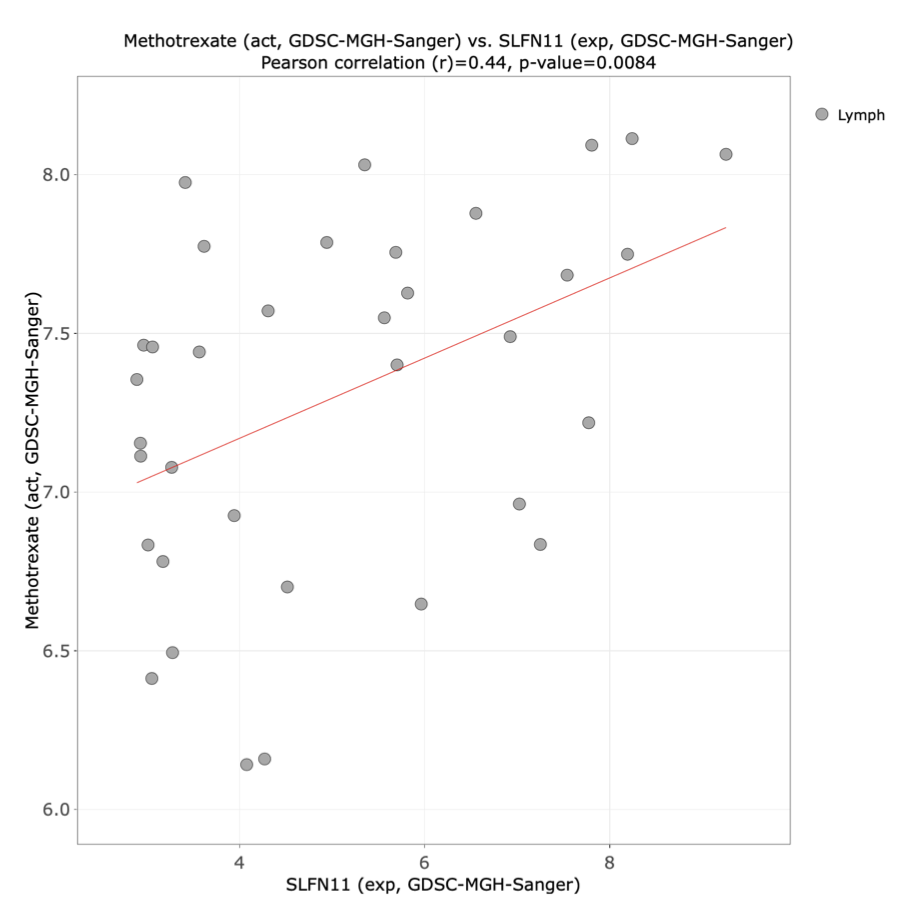

Supplement: Supplementary file 1 [file jcm-11-05548-s001.zip › jcm-1847860-Figure S1.tif]
